# Transplantation of Cultivated Fibroblasts on a Backing of Xenogenic Tissue in the Treatment of Wounds

**DOI:** 10.5195/cajgh.2014.143

**Published:** 2014-12-12

**Authors:** Kabylbek Abugaliyev, Ogay Vyacheslav, Danlybayeva Gaziza, Miras Karzhauov, Akhmadeyeva Zhansaya, Anuar Zhaikenov

**Affiliations:** 1 Republican Scientific Center for Emergency Care, Astana, Kazakhstan; 2 National Center for Biotechnology, Astana, Kazakhstan

**Keywords:** transplantology, tissue engineering, fibroblasts

## Abstract

**Introduction:**

Trophic ulcers are a common health problem, and there are numerous treatment methods. Irreversible damage in the skin, subcutaneous tissue, and fascia with long-term ulcer existence make standard autotransplantation inneffective. Skin grafts are often complicated by partial or complete rejection of skin flaps. The aim of this study was to examine the feasibility of using transplanted cultivated allogenic fibroblasts on the backing of a cellularless xenogenic fabric for wound healing.

**Methods:**

Transplantation of cultured embryonic fibroblasts on a backing of xenogenic tissue was used in the complex treatment of trophic ulcers for stimulation of regenerative processes. Decellularization xenogenic film was previously held. Then allogenic fibroblasts were cultivated on the surface of collagen-elastin matrix. Since 2013, we treated 12 patients with giant ulcers caused by the following: lymphedema (2 patients), vascular disease (3 patients), diabetes (2 patients), after injury (4 patients), and radiation ulcer (1 patient). Dimensions of ulcers were from 150 to 600 cm^2^. Duration of the lower limb ulcers ranged from 8 months to 10 years. For a number of years, all patients were on a complex therapy, which had not resulted in healing wounds. During the operation when excision of granulation tissue was performed, plastic wounds perforated with the ratio 1:2 autoskin. Xenogenic fabric with cultured fibroblasts was applied on top. In this case, xenogenic film protected the skin from drying, created optimal microclimate, and cultured fibroblasts stimulating regeneration and improving engraftment.

**Results:**

The first redress was held on the fifth day. In all cases, the results of engraftment skin grafts achieved maximum possible (100%) and optimal (90%). Complete epithelialization of the cell perforation was seen in five patients on the fifth day and three on seventh day after skin plastics. Average period of inpatient treatment was 20.7 days. All patients were discharged with healed wounds.

**Conclusion:**

Thus, the treatment of trophic ulcers can be successfully solved using advances in biotechnology. Transplantation of cultivated allogenic fibroblasts on a backing of cellularless xenogenic fabric shows good clinical results due to the stimulation of regenerative processes and creates the optimum environment for autotransplants.

